# Role of curcumin in altering gut microbiota for anti-obesity and anti-hyperlipidemic effects

**DOI:** 10.3389/fmicb.2025.1625098

**Published:** 2025-08-20

**Authors:** Jingxi Feng

**Affiliations:** College of Bioscience and Biotechnology, Hunan Agricultural University, Changsha, China

**Keywords:** curcumin, gut microbiota, weight loss, lipid-lowering, obesity

## Abstract

Obesity significantly impacts the health and economy of modern society, and the prevention and treatment of obesity is a key focus of social research. The main reason for obesity is the excessive accumulation of body fat due to metabolic dysfunction, which may result in atherosclerosis, insulin resistance and abnormal lipid metabolism. So far, a number of mechanisms of intestinal flora and plant extracts have been found and applied to the treatment of obesity. However, because of the complexity of gut microbiota composition, it is still not clear which microbiota has a direct relationship with obesity. Curcumin (CUR) has a long-standing and important position in traditional Chinese medicine (TCM). It is a polyphenolic compound derived from the rhizome of *Curcuma longa*. CUR has been widely studied in recent years for its multiple biological activities. In addition to its anti-inflammatory and antioxidant properties, CUR also shows potential in anti-cancer, anti-microbial, and neuroprotective effects. This review aims to systematically synthesize the current evidence on the effects and mechanisms of CUR targeting gut microbiota in obesity treatment, analyze the progress of CUR research in fat reduction and weight loss, and specifically clarify its role in modulating gut microbiota to exert both anti-obesity and anti-hyperlipidemic effects. By doing so, we seek to illuminate the intricate relationship between CUR, gut microbiota, and obesity with associated hyperlipidemia.

## 1 Introduction

Obesity arises when the balance of energy intake, consumption, and storage is disrupted. With the improvement in living standards and increased consumption of high-fat (HF) foods, obesity has become a major public health issue. Obesity is one of the risk factors for hypertension, diabetes, cancer, cardiovascular, and cerebrovascular diseases. Studies have shown that the gut microbiota and the abundance of fat in obese individuals are different from those in healthy individuals, indicating that it is possible to prevent and treat obesity by regulating gut microbiota (Tremaroli and Bäckhed, 2012; Sankararaman et al., 2023; Bagheri et al., 2022). It should be noted that while these observed differences suggest a potential link between gut microbiota regulation and obesity management, the specific mechanistic pathways (e.g., metabolic byproduct signaling, immune modulation, or dietary energy extraction) through which gut microbiota may exert such effects remain incompletely validated in large-scale clinical studies. Most current evidence supporting this “possible” regulatory role is derived from preclinical models or small-scale observational studies, and further rigorous translational research is needed to confirm causality and clarify the precise mechanisms. Gut microbiota, the normal microbes residing in the gut, exhibit a great variety and play a crucial role in digestion, absorption, gut homeostasis, inflammation suppression, immune regulation, and even the central nervous system (CNS).

The gut microbiota’s impact on inflammation is twofold. Firstly, the increased abundance of pathogenic bacteria in obese individuals can elevate serum lipopolysaccharide (LPS) levels, subsequently activating the TLR-4/NF-κB signaling pathway to induce inflammation (Cani et al., 2009). Secondly, the growth of bifidobacteria increases the population of CD4+ CD25+ TReg cells, which can inhibit the inflammatory response mediated by NF-κB (Guo et al., 2015; Engevik et al., 2019). Current obesity treatments include drugs such as norepinephrine, lipase inhibitor orlistat, and dopamine reuptake inhibitor bupropion. These treatments are characterized by multiple effects, multiple targets, and fewer side effects. For instance, orlistat, by inhibiting intestinal lipase, reduces fat absorption while regulating metabolic indices and improves both obesity and metabolic-associated fatty liver disease with favorable safety (Feng et al., 2023; Nikniaz et al., 2023). Curcumin (CUR) may serve as a crucial “target” for reducing “fat flora” and enhancing “weight loss flora.”

## 2 Curcumin: properties and activities

Having outlined the significance of the gut microbiota-obesity axis and the potential role of bioactive compounds in this context, we now focus on CUR—a natural polyphenol with well-documented metabolic regulatory properties—to explore its biological characteristics and relevance to the aforementioned mechanisms. CUR is a natural phenolic antioxidant, which is extracted from the rhizomes of *Curcuma longa*, Curcuma zedoary, *Curcuma* spp., and so on. Its main chain is composed of unsaturated aliphatic and aromatic groups, and it belongs to diketone compounds. CUR accounts for about 3%–6% in these plants and is a very rare pigment with a diketone structure in the plant kingdom. CUR has multiple effects, such as lowering blood lipids, being anti-tumor, anti-inflammation, promoting gallbladder function, and being antioxidant. Among these biological activities, its anticancer potential has been most widely described, thus attracting the attention of scientists around the world. Turmeric, a perennial herb, has been widely used as a dietary spice for centuries and is also used as a traditional natural medicine in Eastern countries. Due to the presence of conjugated double bonds in its chemical structure, CUR can act as an effective electron donor. Studies have indicated that this structural feature serves as a crucial basis for CUR to exhibit various biological activities such as antioxidant properties (Nunes et al., 2024; Dai et al., 2024). In REDOX reactions, this can reduce the production of reactive oxygen species (ROS) (Ayati et al., 2019), Oxidative stress and cell damage can be caused by ROS, and this property makes CUR a potent antioxidant, which is widely used in the food supplement industry (Kunnumakkara et al., 2017).

### 2.1 Biological effects of curcumin

CUR is a powerful and adaptable substance with a broad range of therapeutic applications, and its remarkable effects go beyond conventional medical interventions. It is well-known for its excellent ability to alleviate inflammation, combat free radicals, support the body’s natural defense mechanisms against cancer, and protect against various cardiovascular ailments, thus being a valuable ally in modern medicine (Mondal et al., 2024; Mayo et al., 2024). Obesity is associated with increased levels of interleukin-6 (IL-6) and tumor necrosis factor-α (TNF-α). CUR’s anti-inflammatory action is typically linked to one or more of the following mechanisms: acting on receptors and signaling pathways, controlling how the target tissue reacts to inflammatory mediators, reversing the effects of media on the target tissue, or generating anti-inflammatory mediators (Medzhitov, 2008). CUR has anti-inflammatory properties as it regulates inflammatory signaling pathways and inhibits the synthesis of inflammatory mediators (Peng et al., 2021). CUR has been shown to inhibit the growth of cancer cells in many cancers. Prostate cancer originates from or affects the male prostate epithelium. Considerable research has investigated the anti-cancer influence of CUR on androgen-sensitive prostate cancer cells. For example, a study by Dorai found that the proliferation rate of LNCaP cells decreased to 20%–30% of untreated cells, with a maximal half-maximal inhibitory concentration (IC50) of CUR being 10–20 μM (Dorai et al., 2000). Under the same condition, the level of Bcl-2 binding X (Bax) remained unchanged, but there was significant inhibition on the levels of Bcl-2 and Bcl-xL, showing a higher Bax/Bcl-2 ratio compared to untreated cells. Another example is NF-κB, a pro-inflammatory transcription factor crucial for breast cancer cell growth. It controls the expression of proteins involved in cell signaling pathways and over 500 different genes related to cancer and inflammation. By inhibiting NF-κB interactions, CUR may be useful in cancer treatment. Specifically, by downregulating the NF-κB-inducing gene, CUR can prevent breast cancer cells from proliferating and invading (Liu et al., 2009; Kim et al., 2012).

### 2.2 Edibility and safety of curcumin

CUR, a potent and multifaceted compound derived from the yellow spice turmeric, has been extensively studied not only for its culinary properties but also for its potential health benefits. Its ability to be consumed in various forms such as tea, food, and supplements has made it a popular ingredient in many cuisines worldwide. The United States Food and Drug Administration (FDA) has declared that CUR is a safe substance, and there are no significant risks associated with its use (Cheng et al., 2001; Prasad et al., 2014; Rahimnia et al., 2015).

The wide range of suggested dosages for CUR indicates the need for caution when using this natural compound in daily life. There are numerous dosage recommendations depending on factors such as individual tolerance, intended use, and the particular ailment being treated. In one clinical trial involving healthy individuals who were administered up to 8,000 mg of CUR daily, only negligible amounts of CUR were detected in the blood after a period of regular dosing (Kocaadam and Şanlier, 2017). In contrast, when two separate groups of subjects were given either 10,000 mg or 12,000 mg of CUR respectively, a much more significant concentration was found in their blood serum. Based on these findings, a daily dose of 12,000 mg of CUR is considered safe for healthy individuals since no adverse effects have been observed (Lao et al., 2006; Vareed et al., 2008). However, despite the lack of evidence of harm, further exploration regarding the bioavailability of CUR and the optimal formulation for enhanced absorption and efficacy is still needed.

## 3 Gut microbiota in obesity

With a clear understanding of CUR’s properties, we now shift focus to the gut microbiota itself—exploring its composition, dynamics, and intricate relationship with obesity, which forms the core biological context for CUR’s regulatory effects. There are numerous types of bacteria in the gut, which can be classified into probiotics and pathogens. Probiotics have a close relationship with prebiotics. An imbalance in the gut microbiota can lead to problems such as indigestion and obesity in people. There are three main mechanisms related to obesity: energy metabolism disturbance, inflammation reaction, and brain-gut axis. The composition of gut microbiota is influenced by multiple factors, including genetics, diet, lifestyle, medication (such as antibiotics), environment, and host health. The imbalance of gut microbiota, known as “gut microbiota imbalance,” is associated with the development of inflammatory bowel disease (IBD), obesity, diabetes, cardiovascular diseases, certain cancers, and neuropsychiatric disorders. In recent years, the development of high-throughput sequencing techniques has also promoted the development of gut microbiota diagnosis and treatment strategies (Tang and Cao, 2021). For example, the use of probiotics and prebiotics, as well as fecal microbiota transplant (FMT), is a therapeutic approach developed based on the improved understanding of gut microbiota.

### 3.1 Composition of gut microbiota

Prebiotics, as a type of food supplement, serve as the “nutrition” for probiotics. They are inactive and not affected by human digestive enzymes or gastric acid. Prebiotics can directly reach the stomach and intestines, promote the growth of probiotics in the gut, and inhibit the growth of pathogens. Well-known prebiotics include fructooligosaccharides, galactooligosaccharides, and inulin. Probiotics are various microorganisms that are beneficial to the human body and possess biological activity. They are naturally present in the human body but can also be taken orally. However, the number of living bacteria will be reduced to a certain extent after passing through the mouth, stomach, and small intestine. The most commonly used probiotics are *Bifidobacteria*, *Lactobacillus*, and *Subtilis*. The former two are the main probiotic ingredients in probiotic oral fluids and drinks. *Escherichia coli* is a bacterium that is commonly part of the normal gut microbiota in humans and animals, but some strains can be harmful and serve as important pathogens of childhood diarrhea, often leading to watery stools (Masiga et al., 2022). *Pseudomonas aeruginosa* is another harmful bacterium that often causes clinical symptoms such as pyogenic enteritis, abdominal pain, and diarrhea, which may lead to severe toxicity and infection (Jacobson et al., 2020). Therefore, we should focus on healthy eating to maintain the balance of bacteria in our body and stay healthy.

### 3.2 Mechanism of obesity

Having established the typical composition of gut microbiota, we next explore how dysbiosis—shifts in microbial balance—contributes to the pathogenesis of obesity, focusing on underlying molecular and physiological mechanisms. The accumulation of fat in the body is harmful to health so obesity is a complex, multi-factorial disease. Obesity is a disease that has emerged rapidly and shows no clear signs of disappearing soon. A higher body mass index (BMI) is a risk factor for non-communicable diseases (NCDs), including diabetes, cardiovascular, and musculoskeletal disorders, which can lead to a lower quality of life and reduced longevity (Litwin and Kułaga, 2021).

A persistent energy imbalance between calories consumed and calories expended is one of the main causes of obesity. The mechanism of obesity is complex and involves many aspects, such as heredity, environment, physiology, psychology, and society. Genetic factors play a significant role in obesity. Some genes have been found to be important in weight control, appetite control, and energy metabolism. A person is at a higher risk of becoming obese if there is a family history of obesity. Research on the tissue-specific effects of epigenetic factors in metabolic organs, combined with the latest advances in sequencing techniques, has greatly enhanced our understanding of the mechanisms involved in energy metabolism in obesity. The epigenome, which includes RNA-mediated processes, DNA methylation, and histone modification, is characterized by changes in gene function during mitosis or meiosis without changes in DNA sequences (Gao et al., 2021). High-calorie, HF, and high-carbohydrate diets are major reasons for obesity as they lead to excessive energy consumption. Insufficient physical activity and a sedentary lifestyle are also significant factors contributing to obesity. Moreover, the fast-paced modern life means that many people lack sufficient time or motivation to exercise regularly, resulting in lower energy consumption.

### 3.3 Gut microbiota in lipid and weight regulation

Against the backdrop of obesity mechanisms driven by gut microbiota dysbiosis, we now turn to the positive regulatory role of gut microbiota in lipid metabolism and weight control—key processes that CUR may modulate. The gut microbiota plays a crucial role in regulating body weight and fat content. The microbiota initially convert indigestible carbohydrates into short-chain fatty acids (SCFAs). Subsequently, these SCFAs are either excreted as feces or absorbed via the gut mucosa. What is more, gut microbiota and the brain interact in intricate ways through immunological, endocrine, and neurological processes (Cryan et al., 2019). By means of the “brain-gut axis,” gut microorganisms can impact the host’s CNS, and the CNS in turn influences the composition and structure of gut microbes. Furthermore, the regulation of food intake by the host’s gut microbial population might impact brain function. Subsequent research has demonstrated that gut bacteria may affect a host’s circadian rhythms in relation to nutrition. Increased adiposity may result from disruptions to the circadian rhythm.

**FIGURE 1:**
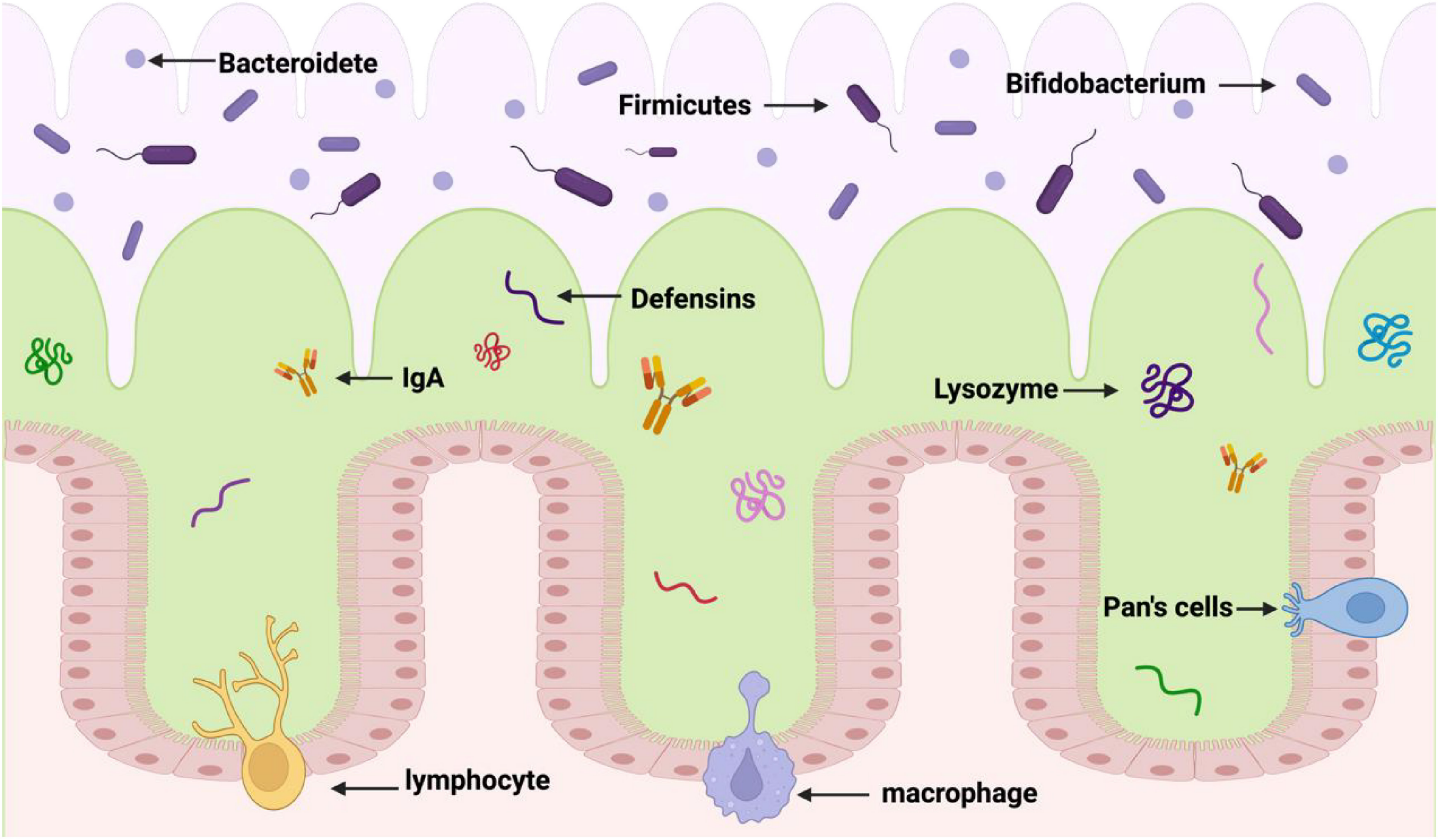
Formation of enteric barrier. The first layer (the purple layer) is the gut cavity. The second layer (the green layer) is the mucus layer of the gut tract, which contains fine bacteria and acts as a barrier against harmful bacteria. The third layer (the pink layer) is the inner mucous layer, which is close to the lower epithelial cells and contains antimicrobial peptides to maintain the function of gut barrier.

These results emphasize how crucial gut microorganisms are for preserving a healthy weight and metabolic balance. Comprehending their role in an individual’s body metabolism might not only improve our understanding of the processes involved in energy expenditure and nutrient intake, but it may also offer suggestions for novel therapeutic approaches. Scientists aim to improve human well-being by investigating the roles of gut microorganisms to learn more about the relationship between nutrition, lifestyle, and illness (Figure 1).

In the present study, the potential remedial role of CUR in reducing insulin resistance and metabolic disturbance in the obese and type 2 diabetic male albino Wistar rat model was investigated. Also, its effect on the expression levels of brain glucose transporter 1 protein (GLUT1) and femoral muscle glucose transporter 4 protein (GLUT4) was examined. It was found that a low dose of streptozotocin (STZ) combined with a HF diet could induce diabetes. CUR was administered intragastrically at a dose of 80 mg/kg BW/day for 8 weeks (Al-Saud, 2020).

In the HF-diet group, obesity, hyperglycemia, hyperinsulinemia, decreased hepatic glycogen content, and severe dyslipidemia were all observed. In the diabetic control group, as measured by the high calculated homeostasis model assessment (HOMA-IR-index score), hyperglycemia and insulin resistance were prominent. Additionally, liver and muscle glycogen contents were reduced, dyslipidemia was present, and liver and pancreatic malondialdehyde levels were significantly elevated. In the control groups with diabetes and obesity, the levels of GLUT1 and GLUT4 were decreasing respectively.

Compared with the diabetic control group, CUR showed a glucose-lowering effect, reduced insulin resistance, dyslipidemia, and malondialdehyde levels in both tissues, and increased the glycogen amounts in the liver and muscles. Moreover, when compared to the diabetic control group, CUR significantly increased GLUT4 gene expression (Al-Saud, 2020).

#### 3.3.1 Energy absorption

The digestive tract cannot efficiently break down complex polysaccharides like cellulose, xylan, and pectin because it lacks polysaccharide hydrolases. Most of the bacteria found in the terminal ileum and colon are anaerobic, meaning they do not make energy through cellular respiration and do not require oxygen to thrive. These anaerobic bacteria are essential for breaking down these polysaccharides because they ferment them into oligosaccharides, which are simpler sugar forms that the colonic flora can further convert into various metabolites. SCFAs, LPS, bile acid, and choline are just a few of the vital substances that the colonic microbiota helps the host produce. These molecules are all essential components of the gut-body communication network. Studies have shown that the effects of SCFAs on the digestive system and overall health and function of the body are extensive. They function as signaling molecules that control metabolism and energy balance, which is crucial for maintaining homeostasis in the gut environment (Nicholson et al., 2012).

**FIGURE 2:**
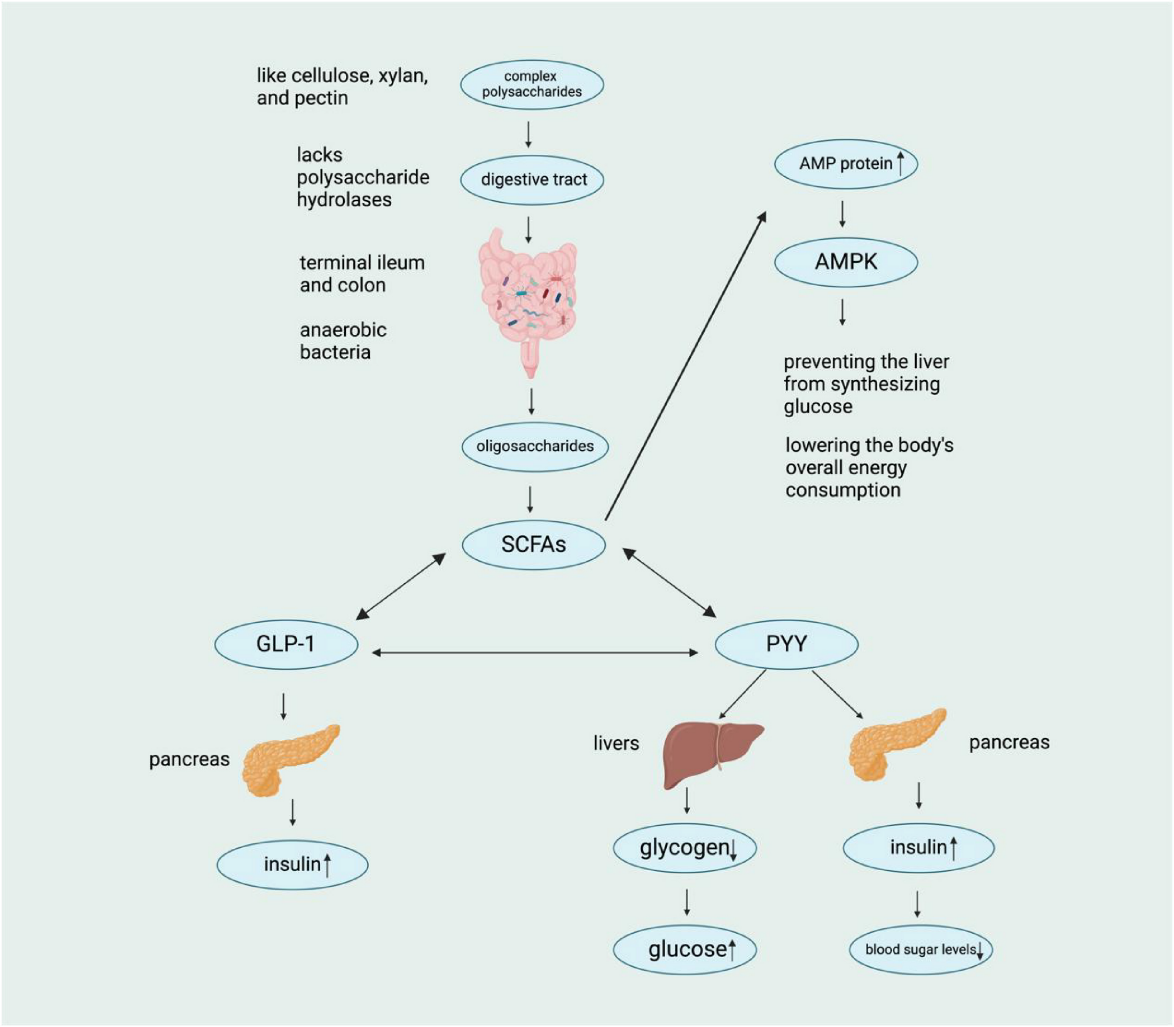
Schematic representation of the metabolic pathway of complex polysaccharides. Complex polysaccharides lacking polysaccharide hydrolases are metabolized by anaerobic bacteria in the digestive tract, leading to the production of SCFAs, which subsequently influence various hormones (GLP-1, PYY, etc.) and ultimately regulate blood sugar levels through complex physiological mechanisms. [In this figure, a downward arrow (↓) indicates a decrease, and an upward arrow (↑) indicates an increase.]

When SCFAs reach their target cells, they stimulate the secretion of two important hormones: glucagon-like peptide 1 (GLP-1) and peptide YY (PYY). The hormone PYY primarily acts to suppress appetite and delay gastric emptying, thereby reducing food intake—a key mechanism for regulating energy balance. In contrast, GLP-1 stimulates pancreatic insulin secretion to lower blood glucose levels, while also inhibiting glucagon release and delaying gastric emptying (Belizário et al., 2018). It is essential to maintain this feedback loop between PYY/GLP-1 and SCFAs to control metabolism and prevent obesity. Moreover, activation of the AMPK signaling pathway, a key regulator of cellular energy homeostasis, further supports metabolic balance by promoting energy-producing processes (e.g., fatty acid oxidation) and inhibiting energy-storing pathways (e.g., denovo lipogenesis). In this regard, regulating AMPK activity may aid in weight reduction by increasing overall energy expenditure (Henning et al., 2018; Hotamisligil, 2006). In this regard, regulating AMPK activity may aid in weight reduction by increasing the body’s overall energy consumption. By understanding these relationships, we can develop strategies that support gut health and improve our overall well-being (Figure 2).

#### 3.3.2 Brain-gut axis

The gut-brain axis (GBA) is a two-way connection between the gut microbiota and the brain. The nerve pathway includes the enteric nervous system (ENS) and the vagus nerve. Meanwhile, endocrine pathways influence the brain’s neuroendocrine system, especially the hypothalamic-pituitary-adrenal (HPA) axis and the immune system (Asadi et al., 2022). Hormones secreted by adipocytes (such as leptin) directly affect the brain’s appetite center (the hypothalamus in particular), thereby regulating appetite and energy balance. Generally, leptin levels increase when the body has sufficient energy, sending a signal to the brain that it is full and should eat less. In contrast, ghrelin levels increase during hunger and stimulate appetite (Obradovic et al., 2021; Jéquier, 2002). Gut endocrine hormones like glucagon-like GLP-1 and pancreatic PYY can reduce gastric emptying, increase satiety, and regulate blood glucose by influencing insulin secretion (Oertel et al., 2024; Dischinger et al., 2019). Disruption of the GBA can cause these hormones to become unregulated, which in turn affects energy metabolism and weight control.

The composition and function of gut microbiota are associated with obesity. Some microbiota may contribute to energy absorption and fat accumulation, while others may help maintain a healthy body weight. The gut microbiota can also indirectly influence appetite and metabolism by affecting the immune system and inflammation. CUR may influence the brain-gut axis through microbiota-derived metabolites (Han et al., 2021). For example, CUR-induced increases in *Bacteroidetes* enhance SCFA production, stimulating enteroendocrine cells to secrete GLP-1, thereby regulating leptin and ghrelin levels and reducing appetite 29 (He et al., 2024). This mechanistic pathway highlights how CUR’s modulation of gut microbiota composition directly impacts host metabolic signaling. Specifically, *Bacteroidetes*-mediated SCFA production (e.g., butyrate and propionate) acts as a key molecular bridge between gut microbiota and central appetite regulation (Panahi et al., 2017).

#### 3.3.3 Biological rhythm

One important mechanism in the relationship between the microbiota and the biological clock the innate lymphoid type 3 cells (ILC3) and signal transducer and activator of transcription-3 (ILC3-STAT3) signaling pathway. A new study shows that the gut microbiota programs the production of histone deacetylase 3 (HDAC3) in the gut epithelium, leading to rhythmic histone acetylation. As a result, the lipid transporter gene, cluster of differentiation-36 (CD36), becomes rhythmic, which promotes obesity and fat absorption (Sonnenberg, 2014). One of the main mechanisms for generating rhythm is the recruitment of histone modifiers to chromatin. Ultimately, the gut microbiota regulates circadian rhythms via HDAC3. It is thought that the circadian rhythm and gut microbiota are regulated by the feeding rhythm. Research indicates that time-limited diets may be able to alleviate the negative effects of HF diets by adjusting the gut microbiota’s circadian rhythm (Ye et al., 2020). Germ-free mice were given the feces of both jet-lagged individuals and those with regular sleep patterns. Mice colonized with feces from jet-lagged individuals developed insulin resistance and obesity. An irregular eating schedule can lead to microbiota abnormalities, which can then cause obesity and insulin resistance (Choi et al., 2021).

Metabolites from the gut microbiota also impact the host’s rhythm system. Bile metabolism is the result of the regular interaction between the host and gut bacteria. The hydrolytic enzymes in the microbial bile salt control circadian rhythm and lipid metabolism (Takahashi et al., 2008). Bile salts are converted by gut bacteria, including *Bacteroides, Bifidobacterium, Ruminococcaceae, Lactobacillus*, and Trichospirillaceae (Wilkinson et al., 2020). SCFAs have a direct effect on liver cell clock gene expression. Acetate or butyrate can control the expression of Period 2 (Per2) and Bmal1 in liver cells. Another study suggested that SCFAs could indirectly regulate the internal clock, as no changes in the peripheral clock were observed in cultured fibroblasts or cultured hepatic sections (Mao et al., 2018).

These mechanisms—energy absorption, brain-gut signaling, and circadian rhythm—establish a foundational framework for understanding how CUR may exert anti-obesity effects through its modulation of gut microbiota composition. This interplay aligns with the core premise that CUR’s regulatory impact on microbial communities can influence key metabolic pathways underlying lipid accumulation and weight regulation, with specific mechanistic links elaborated in the context of subsequent discussions on CUR-gut microbiota interactions.

## 4 Effects of curcumin on gut microbiota

Having clarified how gut microbiota modulates lipid metabolism and weight, we now investigate how CUR—introduced earlier as a bioactive compound with metabolic potential—exerts its effects through regulating gut microbiota composition and function. CUR has been proven to be capable of reducing symptoms of IBD and other enteritis. For instance, the Lopresti et al. (2021) study, in which 500 mg of Curcugen™ was administered once daily for 8 weeks, showed greater improvement in gut discomfort and anxiety among adults with self-reported digestive discomfort. However, compared to placebo, there were no significant changes in gut microbiota and small gut bacterial overgrowth (SIBO). Deeper studies with a larger sample size and more detailed microbiological tests are thus necessary.

Other mechanisms related to CUR’s gut palliative effect is also important. For example, we can examine its effects on gut barrier function, inflammation, neurotransmitter activity, and visceral sensitivity. Furthermore, it has been demonstrated that gut microbiota and metabolism disorders may contribute to the development of Parkinson’s disease (PD), although the underlying mechanism is not fully understood. It has been documented that CUR regulates gut microbiota and has neuroprotective effects on the nervous system. CUR is said to ameliorate alpha-synuclein (α-syn) aggregation, glial cell activation, and the mobility deficit of MPTP-treated mice. According to a serum metabolomics investigation, CUR increased the amounts of creatine, sarcosine, methionine, and tyrosine. It should be noted that the major metabolites (tyrosine, methionine, and creatine) related to motor function and pathological changes were detected in the mouse (Aerococcaceae, Staphylococcaceae, Muribaculaceae, and *Lactobacillus*). After CUR treatment, brain tyrosine and levodopa (Dopa) levels increased rapidly; this increase was associated with the abundance of Lactobacillaceae and Aerococcaceae (Cui et al., 2022). These findings suggest that CUR may prevent PD progression by controlling the gut microbiota-metabolite axis. A key focus of neuroprotection in PD is the CUR-related neuroprotective effects mediated by Lactobacillaceae, Aerococcaceae, and key metabolites such as tyrosine and dopamine.

### 4.1 Modulation of gut microbiota abundance

As is widely known, gut microbiota plays a crucial role in human physiology, and their composition is influenced by a variety of environmental and lifestyle factors (Flandroy et al., 2018; Gentile and Weir, 2018; Rothschild et al., 2018). Any disruption in the gut microbiota spectrum, known as dysbiosis, is significant for disease development. Interestingly, CUR and its metabolites have been demonstrated to have an impact on gut microbiota (Feng et al., 2017; Cotillard et al., 2013). It is important to note that the interaction between CUR and gut microbiota gives rise to two distinct phenomena: CUR directly modulates the gut microbiota, and CUR is transformed by the gut microbiota to generate active metabolites (Lou et al., 2015; Sun et al., 2020). These two effects appear to be essential for CUR’s activity. Based on 16S rRNA sequencing of gut microbiota, the addition of CUR may increase the abundance of *Bacteroides* in the gut microbiota (Yi et al., 2025). Consequently, it is hypothesized that CUR alleviates oxidative damage to the liver by regulating the abundance of the gut microbiota. Specifically, when combined with HF diets, CUR has been shown to significantly reduce the abundance of many previously known inflammatory and diabetic-related species, such as *Ruminococcus* (Lamichhane et al., 2024). Moreover, CUR has successfully decreased 36 potentially harmful bacterial strains that are positively associated with hepatic steatosis (Schneider et al., 2015). These data indicate that CUR could be used to treat fatty hepatic steatosis by targeting the gut microbiota. These findings strongly suggest that the protective effect of CUR is likely due to its capacity to promote a significant shift from pathogenic to beneficial bacteria in the gut. CUR can also promote the transformation of anaerobes, such as *Exiguobacterium*, *Helicobacter*, *Pseudomonas*, *Serratia*, and *Shewanella*. This is partly reversed by the lack of estrogen following oophorectomy (Zhang et al., 2017).

### 4.2 Curcumin for weight loss

Building on CUR’s ability to reshape gut microbiota, we now examine how these microbial changes translate into tangible anti-obesity effects, particularly in the context of weight loss and lipid reduction. There is mounting evidence that CUR treatment can alleviate the altered secretion of proinflammatory mediators associated with obesity and related pathologies. This section compiles and summarizes data from six trials conducted in overweight and obese patients using CUR. CUR (1 g/day) over 8 weeks decreased TNF-α, IL-6, and MCP-1 in male and female patients diagnosed with metabolic syndrome (MS) (Panahi et al., 2016). In a randomized placebo-controlled trial of 60 adolescent girls on a 10-week moderate weight loss diet, the intake of CUR (500 mg/day) resulted in significantly lower levels of hs-CRP and IL-6 compared to the placebo (Saraf-Bank et al., 2019). Additionally, it was demonstrated that CUR could regulate the circulating IL-1β level in 30 volunteers randomly assigned to either CUR (1 g/day) or a placebo for 4 weeks. Treatment with CUR showed a significantly lower level of IL-1β, whereas there was no significant difference in IL-6 and MCP-1 concentrations (Ganjali et al., 2014). In conclusion, CUR supplementation (300 mg/day) for 3 months in type 2 diabetes (T2D) patients led to significantly lower circulating free fatty acid (FFA) levels, which are believed to be a major contributor to obesity and inflammation (Na et al., 2013). CUR exerts notable effects on adipose tissue biology, as demonstrated in key preclinical studies. This study reports that CUR intervention reduces macrophage infiltration in WAT, shifts macrophage polarity toward a more anti-inflammatory profile (evidenced by a higher M2/M1 ratio), and lowers levels of proinflammatory factors such as TNF-α and IL-6 (Song et al., 2018). Additionally, CUR enhances thermogenesis, upregulates UCP1, via PPAR-dependent and -independent mechanisms, latter less characterized. CUR combined with white pepper (0.1% CUR + 0.01% white pepper) in high-fat diet mice for 4 weeks showed no significant changes in body weight or core metabolic markers, but reduced proinflammatory cytokines in subcutaneous adipose tissue with tetrahydrocurcumin accumulation. White pepper may enhance CUR solubility and tissue uptake via piperine, amplifying its adipose actions (Neyrinck et al., 2013) (Table 1).

**TABLE 1:** Effects of different doses and durations of CUR on various biomarkers.

CUR dose	Duration	Effect	Reference
EXPERIENCE			
70 mg/day	8 weeks	↓ TNFα, hs-CRP, and IL-6	Rahmani et al., 2016
1 g/day	8 weeks	↓ TNFα, IL-6, and MCP-1	Panahi et al., 2016
500 mg/day	10 weeks	↓ HS-CRP and IL-6	Saraf-Bank et al., 2019
1 g/day	4 weeks	↓ IL-1β -IL-6 and MCP-1	Ganjali et al., 2014
250 mg/day	8 weeks	↓ FFA	Na et al., 2013
1% CUR/day	18 weeks	↑UCP1	Song et al., 2018
0.1% CUR/day	4 weeks	↓ LPS	Neyrinck et al., 2013

This table presents data showing that varying doses and treatment durations of CUR lead to significant reductions in inflammatory cytokines (e.g., TNFα and IL-6) and other factors (FFA and LPS), as supported by multiple references. [In this table, a downward arrow (↓) indicates a decrease, a horizontal line (-) indicates little or no change.]

### 4.3 Other functions of curcumin

**FIGURE 3:**
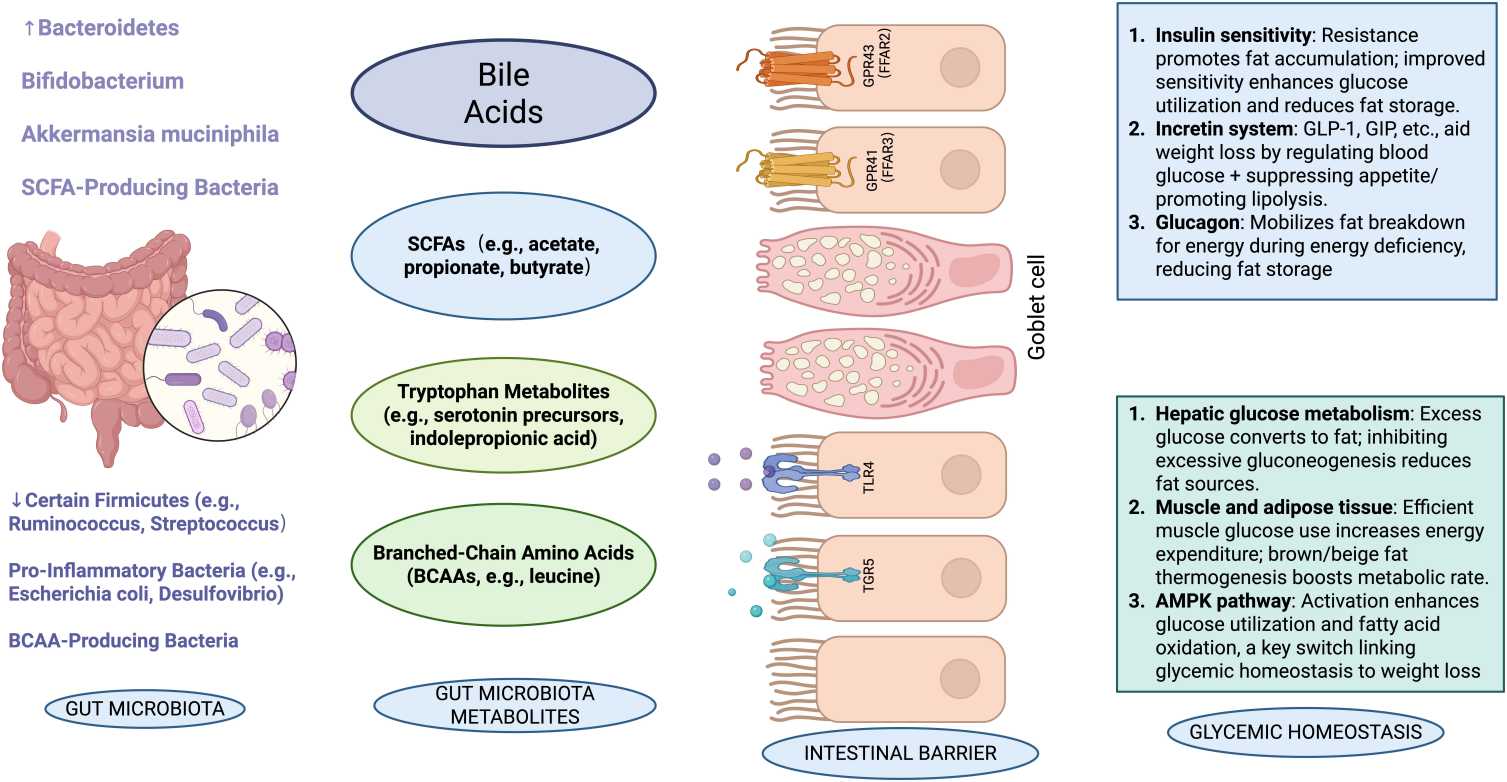
Schematic representation of the interrelationships among gut microbiota, their metabolites, intestinal barrier, and related physiological effects. The diagram illustrates how different microbiota produce metabolites that influence immune pathways, glucose transporters, enterohormones, and ultimately glycemic homeostasis and intestinal integrity. [In this figure, a downward arrow (↓) indicates a decrease, and an upward arrow (↑) indicates an increase.]

Beyond its direct role in weight regulation, CUR exerts broader physiological effects that complement its anti-obesity properties. These include anti-inflammatory, antioxidant, and metabolic regulatory functions that synergize with its gut microbiota-mediated effects. Polyphenols (PP), which are natural bioactive ingredients in fruits and vegetables, are among the richest antioxidants in the human diet. Research has shown that PP may contribute to the improvement of MS, which may be helpful in preventing many chronic conditions, including diabetes, obesity, hypertension, and colon cancer. PP has structural diversity, which affects its bioavailability as it accumulates in the large intestine and is extensively metabolized by the gut microbiota. The gut microbiota converts PP into its metabolites, making them biologically active. Interestingly, gut microbiota not only metabolizes PP but also regulates the composition of the gut microbiota. Thus, a shift from pathogenic to beneficial microbiota can contribute to the improvement of gut health and related diseases (Shabbir et al., 2021). The good news is that CUR is a polyphenolic compound, so it can improve MS, prevent many chronic diseases, and also have beneficial effects on the gut. When administered orally, CUR enters the gut and influences the genetic diversity, richness, and composition of the gut microbiota (He et al., 2024). Shen et al. (2017) has found that CUR has a significant effect on the families of Bacteroidetes, Rikenellaceae, and Prevotellaceae. Another study showed that CUR had a significant effect on weight loss in ovariectomized rats (Zhang et al., 2017). In a recent Al-Saud (2020) study, the administration of CUR (8 weeks, 80 mg/kg/day) demonstrated anti-obesity and anti-diabetes effects in obese Wistar rats while increasing the expression of glucose transporter type 4 (GLUT4). Hepatic and pancreatic glucose-lowering effects, dyslipidemia, decreased insulin resistance, and decreased malondialdehyde were all features of CUR. Koboziev et al. (2020) added CUR to mice susceptible to diet-induced metabolic dysfunction on a HF diet. They found that the animals were protected against osteoarthritis (OA) and obesity, and there was no change in glucose clearance, the size of white adipose cells, or the integrity of the knee. CUR has also been shown to improve gut barrier function through the regulation of intracellular signaling and tight junctions. For example, it reduces the rate at which bacteria spread into the blood, liver, kidney, and spleen (Wang et al., 2017). In one study, it was discovered that CUR could improve the gut barrier and decrease the serum LPS induced by a Western diet (Ghosh et al., 2014) (Figure 3).

Mechanistically, three shared pathways emerge from current research: first, CUR improves the microbial habitat by inhibiting intestinal inflammatory factors (e.g., TNF-α and IL-6); second, its metabolites (e.g., tetrahydrocurcumin) directly regulate the activity of microbial metabolic enzymes; third, it indirectly influences microbiota-host energy metabolism crosstalk via activating the AMPK signaling pathway (Cheng et al., 2023). Notably, the validation of these mechanisms varies across species—for example, the AMPK pathway is well-studied in rodents but lacks sufficient clinical data in humans (He et al., 2024; Xiao et al., 2023; Table 2).

**TABLE 2:** Summary of recent clinical trials investigating CUR efficacy across diverse conditions.

Study	Population studied	Duration	CUR dose	Key results
He et al., 2024	Adults with non-alcoholic simple fatty liver disease (*n* = 80)	24 weeks	500 mg/day (oral, encapsulated formulation)	Reduced hepatic fat, weight, metabolic markers; improved gut microbiota, and TGR5/GLP-1 levels
Xiao et al., 2023	Mice (8 - week - old C57BL/6J)	60 days	100, 200, and 400 mg/kg/day	CUR Ameliorates Age-Induced Tight Junction Impaired in Porcine Sertoli Cells by Inactivating the NLRP3 Inflammasome through the AMPK/SIRT3/SOD2/mtROS Signaling Pathway
Kongkam et al., 2022	132 patients were randomized. 45, 43, and 44 patients were in the curcumin, omeprazole, and placebo groups	28 days and 56 days follow-up	CUR capsules ± placebo/omeprazole	No discernible differences were identified between CUR and omeprazole in their efficacy for the functional treatment of dyspepsia, and no apparent synergistic effect was observed
Shah et al., 2020	74 patients in Radiation-induced oral mucositis (RIOM)	6 weeks	0.1% CUR (freshly prepared using nanoparticles) and 0.15% benzydamine mouthwash	Use of 0.1% CUR mouthwash was able to significantly delay the onset of RIOM
Arabnezhad et al., 2022	A total of 76 subjects (38 in each group)	From 7 days before until 3 days after menstruation for three menstrual cycles	500 mg of curcuminoid+ 5 mg piperine, or placebo daily	CUR supplementation improves vitamin D levels in women with PMS and dysmenorrhea

Summarizes key CUR studies, covering the study population (patients, mice), different intervention duration, dose, and main findings like metabolic changes and symptom relief in various research settings.

### 4.4 Limitations of curcumin

The course of a drug in the body determines its fate. Proper absorption, distribution, metabolism, and excretion of the drug are required for it to produce therapeutic effects in the body. The bioavailability of CUR is limited by its low solubility, poor absorption, rapid elimination, and metabolism. CUR has low solubility in water, and its physicochemical properties are unstable. Only a small amount of CUR is dissolved in the gut tract, limiting its active ingredients. CUR is a highly hydrophobic compound with a solubility of about 11 ng/ml. CUR is very unstable in alkaline conditions, resulting in the degradation of ferulic acid, feruloyl methane, vanillin, and dicyclopentanedione (Vaughn et al., 2016).

Absorption is the process by which a drug enters the bloodstream from the administration site, and CUR is poorly absorbed from the gut tract. When a maximum safe dose of CUR of 12 g/day is administered orally, the terminal level of CUR is only approximately 10 ng/ml at this time (Simental-Mendía et al., 2019). This could be caused by the action of P-glycoprotein and the first-pass effect of the liver. CUR is a substrate of P-glycoprotein, a transmembrane ATP-dependent drug efflux pump, which facilitates the removal of CUR from the gut membrane, thus restricting its permeability. Furthermore, the first-pass effect of the liver results in the metabolism of some CUR in the gut and liver, leading to decreased absorption (Tsuda, 2018). One of the major reasons for low bioavailability is the rapid metabolism of CUR (Chen et al., 2020). After entering the bloodstream, CUR is rapidly metabolized into a stable substance. In phase I metabolism, CUR is reduced to dihydrocurcumin, tetrahydrocurcumin, hexahydrocurcumin, and octahydrocurcumin, and then in phase II, CUR and dimethylcurcumin are metabolized to glucuronide and sulfate, resulting in the formation of non-bioactive glucuronide and sulfate conjugates. Interestingly, 75% of unabsorbed CUR accumulates in the colon (50–200 μM), enabling direct modulation of gut microbiota, which may explain its local bioactivity despite low systemic bioavailability.

CUR research faces several notable limitations that hinder consistent interpretation and translation of findings. Firstly, study designs vary widely—ranging from *in vitro* experiments with isolated cells to animal models and human clinical trials—with little standardization in methodologies, making cross-study comparisons challenging (Singh et al., 2024; Shaban et al., 2025). Secondly, dose discrepancies are striking: animal studies often use extremely high doses to observe effects, which are far higher than the lower doses typically used in human trials, raising questions about translatability. For example, doses range from 1,000 mg/day in elderly populations to 1,600 mg/day in patients with ulcerative colitis, and even up to 4,000 mg/day in some trials (Erol Doğan et al., 2024; Sanmukhani et al., 2014). This heterogeneity hinders the establishment of a unified standard for effective and safe doses in humans. Current evidence suggests that a daily dose of 500–1,000 mg may be effective for metabolic disorders (e.g., non-alcoholic fatty liver disease), while higher doses (1,500–2,000 mg/day) have been used in inflammatory conditions with acceptable safety profiles (Wang et al., 2021). However, adverse effects such as gastrointestinal discomfort may occur at doses exceeding 8,000 mg/day (Qiu et al., 2016). Notably, the U.S. National Institutes of Health (NIH) recommends that daily intake should not exceed 1,500 mg to balance efficacy and safety, but further large-scale randomized controlled trials are needed to validate optimal doses for specific populations (e.g., obese individuals or patients with gut dysbiosis).

Delivery methods also differ significantly, including oral administration (with poor bioavailability due to low solubility), encapsulation in nanoparticles, or parenteral injection, each altering CUR’s absorption, distribution, and interaction with gut microbiota (Roy et al., 2025; Pandey et al., 2025; Olotu et al., 2020). Additionally, study populations exhibit marked heterogeneity, with variations in age, baseline health status, gut microbiota composition, and concurrent medications, all of which can influence CUR’s efficacy and mask consistent trends (Obeid et al., 2023). These inconsistencies underscore the need for more standardized protocols to enhance the reliability and clinical relevance of CUR research.

## 5 Conclusion and prospects

It is evident that CUR has a significant impact on lipid lowering and weight loss, and it also exhibits anti-inflammatory, anti-cancer properties, as well as the ability to improve the abundance and diversity of gut microbiota in obesity. Unfortunately, the low bioavailability of CUR when administered orally may significantly reduce its pharmacological potential and thus restrict its clinical use (Shoba et al., 1998). Additionally, current research on CUR is still limited by unclear mechanisms of action in specific disease contexts, such as its precise regulatory network in obesity-related metabolic disorders, and the lack of large-scale, long-term clinical data to confirm its efficacy and safety in diverse populations.

Despite these challenges, continued research and development in the field of CUR is crucial. Adjuvant preparations produced by combining CUR with piperine, also known as combination therapy, have been shown to inhibit its rapid metabolism and increase its absorption, thereby enhancing CUR levels in serum and improving its bioavailability (Heidari et al., 2023). Other agents, such as quercetin, have been explored, and synergistic effects with doxorubicin (a chemotherapeutic drug) have also been investigated to enhance CUR’s biological activity (Moorthi and Kathiresan, 2013). Therefore, diverse delivery systems, including micelles, liposomes, phospholipid complexes, microemulsions, nanostructured lipid carriers, and biopolymer nanoparticles, have been developed to enhance the bioavailability of CUR. Our understanding of the most effective ways to apply this versatile substance in the fields of medicine and nutrition will advance with scientific knowledge.

Future research should focus on several key directions: First, further exploration of the molecular targets and signaling pathways underlying CUR’s effects in various diseases to establish a more precise theoretical basis for clinical application (Ding et al., 2024). Second, conduction of large-scale, multi-center clinical trials with longer follow-up periods to evaluate the long-term efficacy and safety of CUR, especially in special populations such as the elderly, children, and patients with multiple comorbidities (He et al., 2024). Third, development of more intelligent and targeted delivery systems, such as stimuli-responsive nanoparticles or cell-mediated delivery platforms, to improve CUR’s tissue-specific accumulation and reduce off-target effects (Ding et al., 2024; Yallapu et al., 2015). Fourth, investigation of the synergistic effects of CUR with other bioactive compounds or conventional therapies to maximize therapeutic outcomes while minimizing adverse reactions (Heidari et al., 2023).

In summary, CUR offers many potential health advantages, but its optimal application requires careful consideration and further research. Continued study may lead to safer, more effective, and more accessible methods of using this beneficial plant to enhance human health.
